# Editorial: Putting engineering back in vascular tissue engineering to advance basic science and clinical applications

**DOI:** 10.3389/fcvm.2022.1025465

**Published:** 2022-09-21

**Authors:** Walter L. Murfee, Jessica E. Wagenseil

**Affiliations:** ^1^J. Crayton Pruitt Family Department of Biomedical Engineering, University of Florida, Gainesville, FL, United States; ^2^Department of Mechanical Engineering and Materials Science, Washington University in St. Louis, St. Louis, MO, United States

**Keywords:** vascular, tissue engineering, mechanics, design, blood vessel

Vascular tissue engineering can be characterized as the creation of replacement blood vessels. Over the past 30 years, approaches have incorporated different combinations of extracellular matrix scaffolds, cells, and active bio-chemical cues. Challenged by the goal to recapitulate the complexity of big or small blood vessels, the clinical use of tissue engineered vessel replacements is still limited. With research more often focusing on reductionist materials science or cell biology characterization of vessel-like constructs, an opportunity has re-emerged to apply engineering approaches to guide the next step in vascular tissue engineering. The objective of this Research Topic is to highlight the impact of “engineering,” “engineering design,” and “engineering design verification” in advancing the design of tissue engineered blood vessels (Figure 1). The challenge of integrating cells, scaffolds, and bio-chemical cues into a functional vessel or network of vessels provokes the need to revisit design requirements, the potential role of computational modeling, the opportunity for new manufacturing technologies such as three-dimensional bio-printing, and the application of engineering fundamentals outside the traditional materials science or cell biology tool boxes. By providing examples that span current vascular tissue engineering approaches, the articles in this Research Topic highlight critical questions and opportunities for guiding future therapy development.

**Figure 1:**
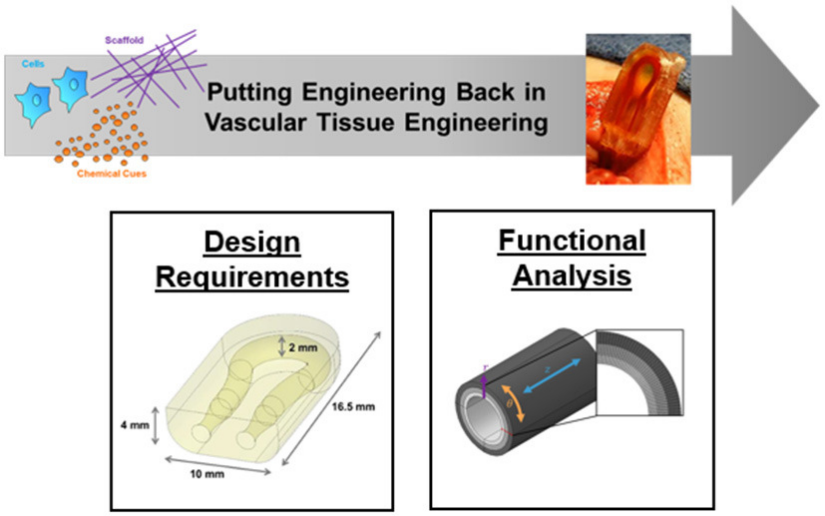
Putting engineering back in vascular tissue engineering to advance basic science and clinical applications. The objective of this Research Topic is to highlight the impact of “engineering,” “engineering design,” and “engineering design verification” in advancing the design of tissue engineering strategies. The challenge of integrating cells, scaffolds, and chemical cues into an engineered vessel or network of vessels provokes the need to revisit the requirements for mimicking real structural, physiological, and mechanical behaviors. Images used for this overview figure were adapted from Galván et al. and Fegan et al.

The overall goal of vascular tissue engineering is design, validation, and eventual clinical transplantation of engineered blood vessels (large or small). The Research Topic serves to remind researchers of the scope of design requirements that need to be considered to mimic the complexity of a tissue replacement. For example, the work by Fegan et al. emphasizes material selection, the design of multi-layer composites, and the importance of mechanical evaluation of synthetic coronary artery grafts to mimic the stress/strain relationships of a biological vessel. Their use of finite

element analysis for evaluating mechanical responses across graft walls motivates reconsideration of the challenges in matching the mechanical properties with multiple cell and matrix constituents. Another requirement for graft design is mimicking the function of a vessel's endothelial cell layer. In their review article, Heng et al. provide a reminder of the decisions to consider when functionalizing materials with surface modifying coating agents to promote graft endothelialization. In the context of engineering microvasculature, the review by Halawani et al. explores various approaches to analogously functionalize extracellular matrix with the goal of assembling endothelial cells into patterned, luminal structures. Methods involving microfluidics, varying scaffold porosity, laser degradation, electrospinning, micro-patterning, and bio-printing are potential options. A question remains—which engineering approach is best? While future research will guide the related therapeutic decision, each method currently offers advantages and disadvantages in the context of their application. Finally, the research example provided by Galván et al. addresses the challenge of transplanting an engineered microvascular network by demonstrating the use of rapid three-dimensional printing of hydrogels for direct-in-line vascular anastomosis.

The complexity associated with engineering vascular tissues remains highlighted by the design goals of mimicking the physiological and mechanical behaviors of a real, multi-cellular environment. In order to push the field closer to clinical applications, we should consider with each tissue engineering approach whether design goals are indeed being met. Often advancements are limited by testing incremental parameter changes in lieu of design iterations and evaluation of the various engineering approaches. We hope that the articles in the Research Topic remind you of the successes and remaining gaps in vascular tissue engineering.

## Author contributions

WM and JW were guest editors for the Research Topic and equally contributed to the writing of this Editorial. Both authors approved the submitted version.

## Funding

Funding for this effort was in part provided by the National Institute of Health (Grant numbers R01AG049821 and R21 HL159501 to WM).

## Conflict of interest

The authors declare that the research was conducted in the absence of any commercial or financial relationships that could be construed as a potential conflict of interest.

## Publisher's note

All claims expressed in this article are solely those of the authors and do not necessarily represent those of their affiliated organizations, or those of the publisher, the editors and the reviewers. Any product that may be evaluated in this article, or claim that may be made by its manufacturer, is not guaranteed or endorsed by the publisher.

